# Isopropyl 2-(5-methyl-3-methyl­sulfinyl-1-benzofuran-2-yl)acetate

**DOI:** 10.1107/S1600536808031796

**Published:** 2008-10-09

**Authors:** Hong Dae Choi, Pil Ja Seo, Byeng Wha Son, Uk Lee

**Affiliations:** a Department of Chemistry, Dongeui University, San 24 Kaya-dong, Busanjin-gu, Busan 614-714, Republic of Korea; b Department of Chemistry, Pukyong National University, 599-1 Daeyeon 3-dong, Nam-gu, Busan 608-737, Republic of Korea

## Abstract

The title compound, C_15_H_18_O_4_S, was prepared by the oxidation of isopropyl 2-(5-methyl-3-methyl­sulfanyl-1-benzofuran-2-yl)acetate with 3-chloro­peroxy­benzoic acid. The crystal structure is stabilized by inter­molecular π–π inter­actions between the benzene rings; the centroid–centroid distance between the adjacent benzene rings (symmetry code: 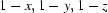) is 3.713 (2) Å. In addition, C—H⋯π and weak inter­molecular C—H⋯O inter­actions are present in the structure.

## Related literature

For the crystal structures of similar 2-(5-methyl-3-methyl­sulfinyl-1-benzofuran-2-yl)acetic acid derivatives, see: Choi *et al.* (2007 ▶, 2008 ▶).
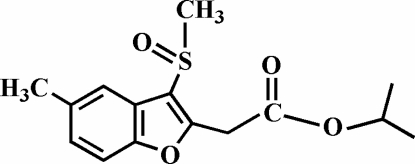


## Experimental

### 

#### Crystal data

C_15_H_18_O_4_S
*M* *_r_* = 294.35
Triclinic, 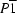

*a* = 8.1829 (5) Å
*b* = 9.7027 (6) Å
*c* = 10.6545 (7) Å
α = 73.057 (1)°
β = 77.463 (1)°
γ = 66.421 (1)°
*V* = 736.76 (8) Å^3^
*Z* = 2
Mo *K*α radiation
μ = 0.23 mm^−1^
*T* = 298 (2) K
0.40 × 0.30 × 0.30 mm

#### Data collection

Bruker SMART CCD diffractometer
Absorption correction: none
5802 measured reflections
2838 independent reflections
2479 reflections with *I* > 2σ(*I*)
*R*_int_ = 0.035

#### Refinement

*R*[*F* ^2^ > 2σ(*F* ^2^)] = 0.036
*wR*(*F* ^2^) = 0.105
*S* = 1.07
2838 reflections
185 parameters
H-atom parameters constrained
Δρ_max_ = 0.22 e Å^−3^
Δρ_min_ = −0.24 e Å^−3^

### 

Data collection: *SMART* (Bruker, 2001 ▶); cell refinement: *SAINT* (Bruker, 2001 ▶); data reduction: *SAINT*; program(s) used to solve structure: *SHELXS97* (Sheldrick, 2008 ▶); program(s) used to refine structure: *SHELXL97* (Sheldrick, 2008 ▶); molecular graphics: *ORTEP-3* (Farrugia, 1997 ▶) and *DIAMOND* (Brandenburg, 1998 ▶); software used to prepare material for publication: *SHELXL97*.

## Supplementary Material

Crystal structure: contains datablocks global, I. DOI: 10.1107/S1600536808031796/fb2111sup1.cif

Structure factors: contains datablocks I. DOI: 10.1107/S1600536808031796/fb2111Isup2.hkl

Additional supplementary materials:  crystallographic information; 3D view; checkCIF report

## Figures and Tables

**Table 1:** Hydrogen-bond geometry (Å, °) *Cg*1 and *Cg*2 are the centroids of the C2–C7 and C1/C2/C7/C8/O1 rings, respectively.

*D*—H⋯*A*	*D*—H	H⋯*A*	*D*⋯*A*	*D*—H⋯*A*
C9—H9*A*⋯O2^i^	0.97	2.38	3.249 (2)	149
C13—H13⋯*Cg*1^ii^	0.98	2.91	3.656 (2)	134
C15—H15*C*⋯*Cg*2^ii^	0.96	2.96	3.837 (2)	152

Symmetry codes: (i) 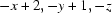; (ii) 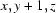.

**Fig. 1.**
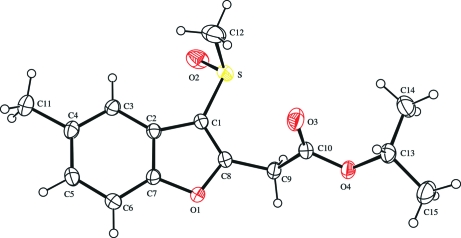
The molecular structure of the title compound, showing the displacement ellipsoids drawn at the 30% probability level.

**Fig. 2.**
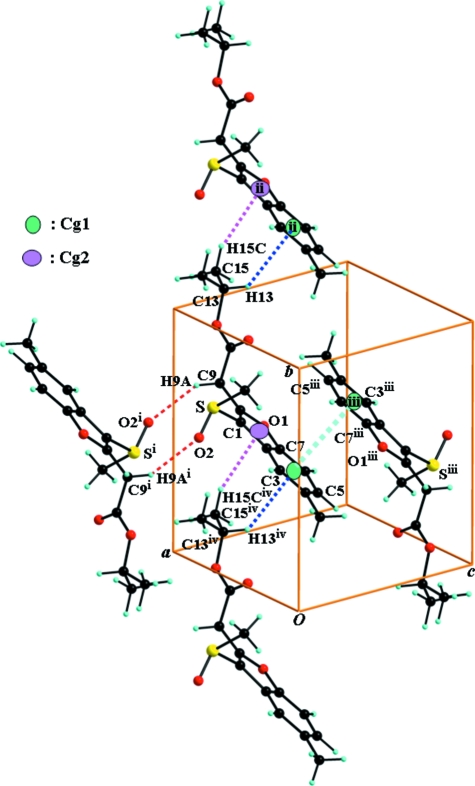
π—π, C—H···π and C—H···O interactions (dotted lines) in the title compound. Cg denotes the ring centroid. [Symmetry codes: (i) -x + 2, -y + 1, -z; (ii) x, y + 1, z; (iii) -x + 1, -y+, -z + 1; (iv) x, y - 1, z.]

## supplementary crystallographic information

### Comment

This work is related to our previous communications on the synthesis and structure of 2-(5-methyl-3-methylsulfinyl-1-benzofuran-2-yl)acetic acid analogues, *viz.* ethyl 2-(5-methyl-3-methylsulfinyl-1-benzofuran-2-yl)acetate (Choi *et al.*, 2007) and methyl 2-(5-methyl-3-methylsulfinyl-1-benzofuran-2-yl)acetate (Choi *et al.*, 2008). Here we report the crystal structure of the title compound, isopropyl 2-(5-methyl-3-methylsulfinyl-1-benzofuran-2-yl)acetate (Fig. 1).

The benzofuran unit is essentially planar, with a mean deviation of 0.004 (1) Å from the least-squares plane defined by the nine constituent atoms. The molecular packing (Fig. 2) is stabilized by π—π electron interactions between the benzofuran rings of the adjacent molecules. The distances between the centroids of the stacked benzene rings are 3.713 (2) Å (symmetry code: 1-x, 1-y, 1-z). The crystal packing is further stabilized by C—H···π electron interactions (Tab. 1). Additionally, intermolecular C—H···O interactions are present in the structure (Tab. 1).

### Experimental

77% 3-chloroperoxybenzoic acid (471 mg, 2.1 mmol) was added in small portions to a stirred solution of isopropyl 2-(5-methyl-3-methylsulfanyl-1-benzofuran-2-yl)acetate (556 mg, 2.0 mmol) in dichloromethane (40 ml) at 273 K. After having been stirred for 3 h at room temperature, the mixture was washed with saturated sodium hydrogencarbonate solution and the organic layer was separated, dried over magnesium sulfate, filtered and concentrated under vacuum. The residue was purified by column chromatography (ethyl acetate) to afford the title compound as a colorless solid [yield 81%, m.p. 403–404 K; *R*_f_ = 0.74 (ethyl acetate)]. Single crystals suitable for X-ray diffraction were prepared by evaporation of a solution of the title compound in ethyl acetate at room temperature. The average crystal size was approximately 1.0 *x* 1.0 *x* 0.5 mm. The crystals are colourless and soluble in polar solvents. Spectroscopic analysis: ^1^H NMR (CDCl_3_, 400 MHz) δ 1.25 (d, J = 6.20 Hz, 6H), 2.46 (s, 3H), 3.08 (s, 3H), 3.99 (s, 2H), 5.00–5.08 (m, 1H), 7.17 (d, J = 8.44 Hz, 1H), 7.39 (d, J = 8.44 Hz, 1H), 7.74 (s, 1H); EI—MS 294 [*M*^+^].

### Refinement

All the hydrogen atoms could have been distinguished in the difference electron density maps. However, all the H atoms were positioned geometrically and refined using a riding model, with C—H = 0.93 Å for the aryl, 0.97 Å for the methylene, 0.98 Å for the methine and 0.96 Å for the methyl H atoms. *U*_iso_(H) = 1.2U_eq_(C) for the aryl, methine and methylene H atoms, and *U*_iso_(H) = 1.5U_eq_(C) for the methyl H atoms.

### Crystal data

**Table:**

C_15_H_18_O_4_S	*Z* = 2
*M**_r_* = 294.35	*F*(000) = 312
Triclinic, *P*1	*D*_x_ = 1.327 Mg m^−^^3^
Hall symbol: -P_1	Melting point = 403–404 K
*a* = 8.1829 (5) Å	Mo *K*α radiation, λ = 0.71073 Å
*b* = 9.7027 (6) Å	Cell parameters from 4030 reflections
*c* = 10.6545 (7) Å	θ = 2.4–28.2°
α = 73.057 (1)°	µ = 0.23 mm^−^^1^
β = 77.463 (1)°	*T* = 298 K
γ = 66.421 (1)°	Block, colourless
*V* = 736.76 (8) Å^3^	0.40 × 0.30 × 0.30 mm

### Data collection

**Table:**

Bruker SMART CCD diffractometer	2479 reflections with *I* > 2σ(*I*)
Radiation source: fine-focus sealed tube	*R*_int_ = 0.035
graphite	θ_max_ = 26.0°, θ_min_ = 2.7°
Detector resolution: 10.0 pixels mm^-1^	*h* = −10→10
φ and ω scans	*k* = −11→11
5802 measured reflections	*l* = −13→13
2838 independent reflections	

### Refinement

**Table:**

Refinement on *F*^2^	Primary atom site location: structure-invariant direct methods
Least-squares matrix: full	Secondary atom site location: difference Fourier map
*R*[*F*^2^ > 2σ(*F*^2^)] = 0.036	Hydrogen site location: difference Fourier map
*wR*(*F*^2^) = 0.105	H-atom parameters constrained
*S* = 1.07	*w* = 1/[σ^2^(*F*_o_^2^) + (0.0509*P*)^2^ + 0.2101*P*] where *P* = (*F*_o_^2^ + 2*F*_c_^2^)/3
2838 reflections	(Δ/σ)_max_ < 0.001
185 parameters	Δρ_max_ = 0.22 e Å^−^^3^
0 restraints	Δρ_min_ = −0.24 e Å^−^^3^
68 constraints	

### Special details

**Table:**

Geometry. All e.s.d.'s (except the e.s.d. in the dihedral angle between two l.s. planes) are estimated using the full covariance matrix. The cell e.s.d.'s are taken into account individually in the estimation of e.s.d.'s in distances, angles and torsion angles; correlations between e.s.d.'s in cell parameters are only used when they are defined by crystal symmetry. An approximate (isotropic) treatment of cell e.s.d.'s is used for estimating e.s.d.'s involving l.s. planes.
Refinement. Refinement of *F*^2^ against ALL reflections. The weighted *R*-factor *wR* and goodness of fit *S* are based on *F*^2^, conventional *R*-factors *R* are based on *F*, with *F* set to zero for negative *F*^2^. The threshold expression of *F*^2^ > σ(*F*^2^) is used only for calculating *R*-factors(gt) *etc*. and is not relevant to the choice of reflections for refinement. *R*-factors based on *F*^2^ are statistically about twice as large as those based on *F*, and *R*- factors based on ALL data will be even larger.

### Fractional atomic coordinates and isotropic or equivalent isotropic displacement parameters (Å^2^)

**Table:**

	*x*	*y*	*z*	*U*_iso_*/*U*_eq_	
S	0.74200 (6)	0.64759 (5)	0.04151 (4)	0.04706 (16)	
O1	0.84827 (15)	0.47478 (12)	0.40980 (11)	0.0378 (3)	
O2	0.7528 (2)	0.53592 (18)	−0.03283 (14)	0.0705 (4)	
O3	0.73496 (17)	0.88857 (15)	0.23749 (16)	0.0603 (4)	
O4	1.00780 (15)	0.88109 (12)	0.25158 (12)	0.0422 (3)	
C1	0.7543 (2)	0.55223 (18)	0.20899 (15)	0.0356 (3)	
C2	0.6619 (2)	0.45172 (16)	0.29204 (15)	0.0336 (3)	
C3	0.5364 (2)	0.39518 (18)	0.27713 (16)	0.0380 (4)	
H3	0.4913	0.4233	0.1967	0.046*	
C4	0.4806 (2)	0.29641 (19)	0.38454 (17)	0.0405 (4)	
C5	0.5499 (2)	0.25528 (19)	0.50517 (17)	0.0424 (4)	
H5	0.5109	0.1888	0.5761	0.051*	
C6	0.6736 (2)	0.30967 (18)	0.52246 (16)	0.0401 (4)	
H6	0.7189	0.2818	0.6027	0.048*	
C7	0.7260 (2)	0.40767 (17)	0.41396 (15)	0.0343 (3)	
C8	0.8622 (2)	0.56095 (18)	0.28352 (15)	0.0361 (3)	
C9	0.9868 (2)	0.64659 (18)	0.25585 (18)	0.0405 (4)	
H9A	1.0607	0.6321	0.1728	0.049*	
H9B	1.0657	0.6029	0.3247	0.049*	
C10	0.8915 (2)	0.81825 (18)	0.24831 (16)	0.0384 (4)	
C11	0.3469 (3)	0.2312 (3)	0.3719 (2)	0.0598 (5)	
H11A	0.2959	0.2846	0.2902	0.090*	
H11B	0.2533	0.2443	0.4443	0.090*	
H11C	0.4066	0.1232	0.3731	0.090*	
C12	0.5114 (3)	0.7722 (3)	0.0561 (2)	0.0717 (6)	
H12A	0.4814	0.8392	−0.0284	0.108*	
H12B	0.4926	0.8332	0.1182	0.108*	
H12C	0.4367	0.7112	0.0865	0.108*	
C13	0.9392 (2)	1.04906 (18)	0.24297 (18)	0.0426 (4)	
H13	0.8239	1.0791	0.2982	0.051*	
C14	0.9158 (3)	1.1356 (2)	0.1023 (2)	0.0603 (5)	
H14A	0.8323	1.1104	0.0707	0.090*	
H14B	1.0294	1.1071	0.0488	0.090*	
H14C	0.8708	1.2446	0.0974	0.090*	
C15	1.0762 (3)	1.0762 (2)	0.2963 (3)	0.0692 (6)	
H15A	1.1907	1.0407	0.2453	0.104*	
H15B	1.0850	1.0206	0.3868	0.104*	
H15C	1.0407	1.1846	0.2910	0.104*	

### Atomic displacement parameters (Å^2^)

**Table:**

	*U*^11^	*U*^22^	*U*^33^	*U*^12^	*U*^13^	*U*^23^
S	0.0572 (3)	0.0469 (3)	0.0365 (2)	−0.0241 (2)	−0.00906 (19)	0.00182 (17)
O1	0.0416 (6)	0.0381 (6)	0.0392 (6)	−0.0204 (5)	−0.0113 (5)	−0.0035 (5)
O2	0.0994 (12)	0.0694 (9)	0.0444 (8)	−0.0262 (9)	−0.0159 (8)	−0.0158 (7)
O3	0.0408 (7)	0.0449 (7)	0.0966 (11)	−0.0190 (6)	−0.0103 (7)	−0.0113 (7)
O4	0.0420 (6)	0.0328 (6)	0.0568 (7)	−0.0177 (5)	−0.0114 (5)	−0.0075 (5)
C1	0.0406 (8)	0.0324 (7)	0.0355 (8)	−0.0163 (7)	−0.0064 (6)	−0.0040 (6)
C2	0.0373 (8)	0.0292 (7)	0.0352 (8)	−0.0128 (6)	−0.0055 (6)	−0.0067 (6)
C3	0.0411 (9)	0.0370 (8)	0.0415 (8)	−0.0167 (7)	−0.0096 (7)	−0.0098 (7)
C4	0.0394 (9)	0.0362 (8)	0.0512 (10)	−0.0175 (7)	−0.0047 (7)	−0.0124 (7)
C5	0.0458 (9)	0.0356 (8)	0.0454 (9)	−0.0205 (7)	−0.0016 (7)	−0.0031 (7)
C6	0.0449 (9)	0.0377 (8)	0.0375 (8)	−0.0165 (7)	−0.0095 (7)	−0.0024 (6)
C7	0.0357 (8)	0.0304 (7)	0.0394 (8)	−0.0140 (6)	−0.0076 (6)	−0.0063 (6)
C8	0.0395 (8)	0.0312 (7)	0.0386 (8)	−0.0159 (6)	−0.0057 (6)	−0.0040 (6)
C9	0.0396 (9)	0.0376 (8)	0.0490 (9)	−0.0200 (7)	−0.0070 (7)	−0.0068 (7)
C10	0.0394 (9)	0.0387 (8)	0.0397 (8)	−0.0206 (7)	−0.0038 (7)	−0.0038 (6)
C11	0.0605 (12)	0.0615 (12)	0.0731 (13)	−0.0393 (10)	−0.0128 (10)	−0.0095 (10)
C12	0.0665 (14)	0.0616 (13)	0.0692 (14)	−0.0084 (11)	−0.0246 (11)	0.0023 (11)
C13	0.0444 (9)	0.0327 (8)	0.0540 (10)	−0.0160 (7)	−0.0051 (8)	−0.0119 (7)
C14	0.0797 (15)	0.0395 (10)	0.0607 (12)	−0.0235 (10)	−0.0124 (10)	−0.0039 (8)
C15	0.0745 (15)	0.0483 (11)	0.1006 (18)	−0.0231 (11)	−0.0311 (13)	−0.0232 (11)

### Geometric parameters (Å, °)

**Table:**

S—O2	1.4839 (15)	C8—C9	1.490 (2)
S—C1	1.7583 (16)	C9—C10	1.516 (2)
S—C12	1.790 (2)	C9—H9A	0.9700
O1—C8	1.3698 (18)	C9—H9B	0.9700
O1—C7	1.3830 (18)	C11—H11A	0.9600
O3—C10	1.199 (2)	C11—H11B	0.9600
O4—C10	1.3305 (19)	C11—H11C	0.9600
O4—C13	1.4774 (19)	C12—H12A	0.9600
C1—C8	1.349 (2)	C12—H12B	0.9600
C1—C2	1.450 (2)	C12—H12C	0.9600
C2—C7	1.392 (2)	C13—C15	1.500 (3)
C2—C3	1.397 (2)	C13—C14	1.500 (3)
C3—C4	1.386 (2)	C13—H13	0.9800
C3—H3	0.9300	C14—H14A	0.9600
C4—C5	1.403 (2)	C14—H14B	0.9600
C4—C11	1.511 (2)	C14—H14C	0.9600
C5—C6	1.379 (2)	C15—H15A	0.9600
C5—H5	0.9300	C15—H15B	0.9600
C6—C7	1.376 (2)	C15—H15C	0.9600
C6—H6	0.9300		
			
O2—S—C1	108.11 (8)	H9A—C9—H9B	107.7
O2—S—C12	106.21 (11)	O3—C10—O4	124.63 (15)
C1—S—C12	97.74 (9)	O3—C10—C9	125.20 (14)
C8—O1—C7	106.03 (11)	O4—C10—C9	110.14 (13)
C10—O4—C13	117.80 (13)	C4—C11—H11A	109.5
C8—C1—C2	107.31 (13)	C4—C11—H11B	109.5
C8—C1—S	123.03 (12)	H11A—C11—H11B	109.5
C2—C1—S	129.65 (12)	C4—C11—H11C	109.5
C7—C2—C3	119.08 (14)	H11A—C11—H11C	109.5
C7—C2—C1	104.42 (13)	H11B—C11—H11C	109.5
C3—C2—C1	136.50 (15)	S—C12—H12A	109.5
C4—C3—C2	118.66 (15)	S—C12—H12B	109.5
C4—C3—H3	120.7	H12A—C12—H12B	109.5
C2—C3—H3	120.7	S—C12—H12C	109.5
C3—C4—C5	119.95 (15)	H12A—C12—H12C	109.5
C3—C4—C11	120.26 (16)	H12B—C12—H12C	109.5
C5—C4—C11	119.79 (16)	O4—C13—C15	105.45 (14)
C6—C5—C4	122.50 (15)	O4—C13—C14	109.85 (14)
C6—C5—H5	118.7	C15—C13—C14	112.29 (17)
C4—C5—H5	118.7	O4—C13—H13	109.7
C7—C6—C5	116.08 (15)	C15—C13—H13	109.7
C7—C6—H6	122.0	C14—C13—H13	109.7
C5—C6—H6	122.0	C13—C14—H14A	109.5
C6—C7—O1	125.47 (14)	C13—C14—H14B	109.5
C6—C7—C2	123.73 (14)	H14A—C14—H14B	109.5
O1—C7—C2	110.80 (13)	C13—C14—H14C	109.5
C1—C8—O1	111.44 (13)	H14A—C14—H14C	109.5
C1—C8—C9	132.89 (15)	H14B—C14—H14C	109.5
O1—C8—C9	115.66 (13)	C13—C15—H15A	109.5
C8—C9—C10	113.54 (14)	C13—C15—H15B	109.5
C8—C9—H9A	108.9	H15A—C15—H15B	109.5
C10—C9—H9A	108.9	C13—C15—H15C	109.5
C8—C9—H9B	108.9	H15A—C15—H15C	109.5
C10—C9—H9B	108.9	H15B—C15—H15C	109.5
			
O2—S—C1—C8	133.07 (16)	C3—C2—C7—C6	0.4 (2)
C12—S—C1—C8	−117.00 (17)	C1—C2—C7—C6	−179.35 (15)
O2—S—C1—C2	−45.61 (17)	C3—C2—C7—O1	−179.98 (13)
C12—S—C1—C2	64.32 (17)	C1—C2—C7—O1	0.30 (17)
C8—C1—C2—C7	0.00 (18)	C2—C1—C8—O1	−0.30 (19)
S—C1—C2—C7	178.83 (13)	S—C1—C8—O1	−179.23 (11)
C8—C1—C2—C3	−179.65 (18)	C2—C1—C8—C9	−178.84 (17)
S—C1—C2—C3	−0.8 (3)	S—C1—C8—C9	2.2 (3)
C7—C2—C3—C4	−0.3 (2)	C7—O1—C8—C1	0.48 (18)
C1—C2—C3—C4	179.36 (17)	C7—O1—C8—C9	179.29 (13)
C2—C3—C4—C5	0.1 (2)	C1—C8—C9—C10	74.3 (2)
C2—C3—C4—C11	−179.20 (16)	O1—C8—C9—C10	−104.20 (16)
C3—C4—C5—C6	0.0 (3)	C13—O4—C10—O3	0.8 (2)
C11—C4—C5—C6	179.31 (17)	C13—O4—C10—C9	179.02 (13)
C4—C5—C6—C7	0.1 (3)	C8—C9—C10—O3	−14.9 (2)
C5—C6—C7—O1	−179.86 (15)	C8—C9—C10—O4	166.85 (14)
C5—C6—C7—C2	−0.3 (2)	C10—O4—C13—C15	160.41 (17)
C8—O1—C7—C6	179.16 (15)	C10—O4—C13—C14	−78.39 (19)
C8—O1—C7—C2	−0.48 (17)		

### Hydrogen-bond geometry (Å, °)

**Table:**

*D*—H···*A*	*D*—H	H···*A*	*D*···*A*	*D*—H···*A*
C9—H9A···O2^i^	0.97	2.38	3.249 (2)	149
C13—H13···Cg1^ii^	0.98	2.91	3.656 (2)	134
C15—H15C···Cg2^ii^	0.96	2.96	3.837 (2)	152

Symmetry codes: (i) −*x*+2, −*y*+1, −*z*; (ii) *x*, *y*+1, *z*.
